# Inhibition of the Lectin Pathway of Complement Activation Reduces Acute Respiratory Distress Syndrome Severity in a Mouse Model of SARS-CoV-2 Infection

**DOI:** 10.1093/infdis/jiad462

**Published:** 2023-10-25

**Authors:** Youssif M Ali, George W Carnell, Stefano Fumagalli, Domenico Mercurio, Serena Seminara, Nicholas J Lynch, Priyanka Khatri, Chanuka H Arachchilage, Luca Mascheroni, Clemens Kaminski, Charlotte L George, Hazel Stewart, Munehisa Yabuki, Gregory Demopulos, Jonathan L Heeney, Wilhelm Schwaeble

**Affiliations:** Department of Veterinary Medicine, School of Biological Sciences, University of Cambridge, Cambridge CB3 0ES, UK; Department of Microbiology and Immunology, Faculty of Pharmacy, Mansoura University, Mansoura 35516, Egypt; Department of Veterinary Medicine, School of Biological Sciences, University of Cambridge, Cambridge CB3 0ES, UK; Department of Acute Brain and Cardiovascular Injury, Laboratory of Stroke and Vascular Dysfunctions, Mario Negri IRCCS, via Mario Negri 2, 20156 Milano, Italy; Department of Acute Brain and Cardiovascular Injury, Laboratory of Stroke and Vascular Dysfunctions, Mario Negri IRCCS, via Mario Negri 2, 20156 Milano, Italy; Department of Acute Brain and Cardiovascular Injury, Laboratory of Stroke and Vascular Dysfunctions, Mario Negri IRCCS, via Mario Negri 2, 20156 Milano, Italy; Department of Veterinary Medicine, School of Biological Sciences, University of Cambridge, Cambridge CB3 0ES, UK; Department of Veterinary Medicine, School of Biological Sciences, University of Cambridge, Cambridge CB3 0ES, UK; Department of Veterinary Medicine, School of Biological Sciences, University of Cambridge, Cambridge CB3 0ES, UK; Department of Chemical Engineering and Biotechnology, University of Cambridge, Cambridge CB3 0AS, UK; Department of Chemical Engineering and Biotechnology, University of Cambridge, Cambridge CB3 0AS, UK; Department of Veterinary Medicine, School of Biological Sciences, University of Cambridge, Cambridge CB3 0ES, UK; Department of Pathology, University of Cambridge, Cambridge CB2 1QP, UK; Omeros Corporation, Seattle, Washington 98119, USA; Omeros Corporation, Seattle, Washington 98119, USA; Department of Veterinary Medicine, School of Biological Sciences, University of Cambridge, Cambridge CB3 0ES, UK; Department of Veterinary Medicine, School of Biological Sciences, University of Cambridge, Cambridge CB3 0ES, UK

**Keywords:** ARDS, complement system, COVID-19, lectin pathway, SARS-CoV-2

## Abstract

Most patients with COVID-19 in the intensive care unit develop an acute respiratory distress syndrome characterized by severe hypoxemia, decreased lung compliance, and high vascular permeability. Activation of the complement system is a hallmark of moderate and severe COVID-19, with abundant deposition of complement proteins in inflamed tissue and on the endothelium during COVID-19. Using a transgenic mouse model of SARS-CoV-2 infection, we assessed the therapeutic utility of an inhibitory antibody (HG4) targeting MASP-2, a key enzyme in the lectin pathway. Treatment of infected mice with HG4 reduced the disease severity score and improved survival vs mice that received an isotype control antibody. Administration of HG4 significantly reduced the lung injury score, including alveolar inflammatory cell infiltration, alveolar edema, and alveolar hemorrhage. The ameliorating effect of MASP-2 inhibition on the severity of COVID-19 pathology is reflected by a significant reduction in the proinflammatory activation of brain microglia in HG4-treated mice.

## Teaser

The critical contribution of the lectin activation pathway of complement to life-threatening systemic inflammation is shown in a murine model of SARS-CoV-2 infection. Inhibition of the lectin pathway key enzyme MASP-2 protects from the fatal outcome of severe inflammatory disease.

The outbreak of COVID-19, a pandemic respiratory disease caused by SARS-CoV-2, is responsible for critical health problems around the world, with a high rate of mortality, especially among the elderly and patients with underlying medical conditions [1]. Variants of early-lineage SARS-CoV-2 are circulating worldwide and accruing mutations that increase its ability to infect susceptible and vaccinated hosts, as well as to escape vaccine or infection-derived immunity [2–4]. SARS-CoV-2 is transmitted by respiratory droplets and aerosols through direct and indirect contact with patients who are infected. Patients may show different degrees of disease severity, including mild, moderate, severe, and critical illness. In mild to moderate cases, patients experience fever, headache, myalgia, cough, and shortness of breath. Patients with severe disease experience hypoxia, shortening of breath, and bilateral radiographic opacities occurring several days postinfection [5]. These symptoms may advance quickly to acute respiratory distress syndrome (ARDS) that requires respiratory support. ARDS is an acute lung injury characterized by severe inflammation, pulmonary vascular leakage, and respiratory failure [6].

Patients who are severely ill with COVID-19 have various neurologic symptoms, such as headache, ischemic stroke, seizures, delirium, anosmia, and encephalopathy [7–12]. Other severe manifestations have been reported, including cortical signal alteration, loss of white matter, and axonal injury [13, 14].

Microglial cells are brain-resident macrophages that play a vital role in the differentiation and maturation of neuronal cells by controlling the process of axonal pruning and by maintaining the homeostasis of central nervous system (CNS) tissue through the removal of apoptotic and injured cells and cellular debris [15]. They also undergo visible morphologic changes depending on their state and degree of activation [16, 17]. Homeostatic microglia are easily recognized by their thin and ramified shape, while reactive microglia are characterized by a hypertrophic shape with greater size and increased ramification. Microglia contribute to the clearance of pathogens and debris, tissue repair, and the release of cytokines and chemokines to attract peripheral immune cells to the infected or injured brain tissue [18]. The morphologic changes that discriminate homeostatic microglia from reactive microglia can be used as a temporospatial sensor to monitor proinflammatory events in the CNS in response to systemic or localized injury or to viral and bacterial infections.

Complement is an important component of innate immunity that participates in the recognition, opsonization, and killing of different pathogens, including viruses. Complement is activated via 3 pathways: the classical pathway (CP), the lectin pathway (LP), and the alternative pathway (AP). The CP is activated when the recognition subcomponent C1q binds to immune complexes, a step that catalyses the autoactivation of the CP-specific serine proteases C1r and C1s. The LP is activated through binding of the recognition subcomponents to their ligands, which include mannan-binding lectin (MBL), collections, as well as ficolins. LP recognition subcomponents bind to dimers of any of the 3 LP serin proteases, termed *MBL-associated serine proteases* (ie, MASP-1, MASP-2, and MASP-3). Of those, only MASP-2 can cleave complement C4 and C4b-bound C2 to form the C3 convertase C4bC2a [19]. The AP is activated via spontaneous hydrolysis of C3 to form C3(H_2_O) and acts as an amplification loop for the CP and the LP [20]. Regardless of the initial activation step, activation of the complement cascade leads to cleavage of C3 and subsequently cleavage of C5, generating the anaphylatoxins C3a and C5a. Furthermore, activation triggers the formation and deposition of the membrane attack complex comprising C5b-9 [21]. The complement activation products C3a and C5a aggravate the state of inflammation, as they serve as anaphylatoxins and contribute to leukocyte infiltration through chemotaxis [22, 23].

Complement activation was reported to be associated with development of ARDS and respiratory failure during viral pneumonia [24]. The involvement of complement-mediated pathology and lung injury during SARS-CoV-2 infection was revealed by a histopathology study of postmortem biopsies taken from patients with COVID-19. The presence of thrombotic microangiopathies and the deposition of complement activation products in injured tissues and organs, including C5b-9, C3d, C4d, and MASP-2, implied the involvement of LP and CP activation in severe COVID-19 [25]. Several reports showed elevation of complement C5a and soluble C5b-9 in sera from patients with COVID-19, indicating activation of complement during SARS-CoV-2 infection [22, 23, 26, 27]. Such findings have led to the trial of complement therapeutics in the treatment of patients who are critically ill with COVID-19. Narsoplimab (OMS721)—a human immunoglobulin gamma 4 (IgG4) monoclonal antibody against MASP-2 that inhibits LP activity—demonstrated a significant benefit in the treatment of patients who were critically ill with COVID-19 in a phase 2 open-label clinical study [28].

Despite intensive research, there are limited options for therapeutic interventions in patients with COVID-19 ARDS [29]. Here we present a mouse model of COVID-19 and show that inhibition of MASP-2 reduces the severity of SARS-CoV-2–driven ARDS, limits the proinflammatory activation of microglia in CNS tissue, and increases survival.

## METHODS

### Ethics Statement

Animal procedures were approved by the Animal Welfare and Ethical Review Body of the University of Cambridge under Home Office project licence P8143424B.

### Cells, Viruses, and Anti-MASP-2 (HG4) Antibody

B.1.351 virus was grown in Vero + hACE2 + TMPRSS2 cells under standard conditions as previously described [30]. The inhibitory anti–MASP-2 antibody HG4 (modified for improved LP inhibition in mice) is derived from the MASP-2 inhibitory monoclonal antibody narsoplimab (OMS721) [16] and was provided by the OMEROS Corporation.

### Infection of Mice With SARS-CoV-2

Groups of 12-week-old k18-hACE2 mice (B6.Cg-Tg[k18-ACE2]2Prlmn/J; JAX) were held in animal containment level 3 facilities in hermetically sealed isocages. Mice were infected with 10^4^ plaque-forming units of SARS-CoV-2 β (B.1.351) variant of concern by intranasal administration in 40 µL of phosphate-buffered saline (PBS) under light anesthesia. Cages were blinded, and mice were closely observed and carefully assessed by 3 independent evaluators. Animals were checked for clinical symptoms, such as hunched posture, piloerection, starry coat, lack of grooming, reduction in the mouse activity, difficult breathing, and nonresponsiveness to external stimuli. Mice approaching the humane end point were euthanized.

### Detection of SARS-CoV-2 in Lung and Brain Tissues From Infected Mice

Lung and brain tissues from infected animals were crushed through a 70-µm cell strainer in 1 mL of PBS and centrifuged for 10 minutes at 1500*g* to clarify cell debris. Viral RNA was extracted with the QIAamp Viral RNA Mini Kit (QIAGEN) following the manufacturer's instructions. Viral RNA was reverse transcribed, and viral copy numbers were normalized to copies per gram of lung or brain tissues tissue as previously described [30].

### Detection of Specific Antibodies Against SARS-CoV-2

The specific antibody titers against viral spike protein (SP) or nucleocapsid protein (NP) were measured in the blood of the infected and noninfected mice as previously described [31].

### Tissue Processing

Lungs and brains were collected from euthanized mice and fixed with 10% neutral-buffered formalin for 48 hours at room temperature; then, formalin was changed to 70% ethanol for another 24 hours. Afterward, tissues were embedded in paraffin wax for histology and immunohistochemistry [32]. Lung injury score was calculated as previously described [33]. Briefly, lung sections were blinded and examined by 3 independent evaluators for leukocyte infiltration, thickening of interstitial membranes, acute intra-alveolar edema, deposition of fibrin, diffuse alveolar damage, and alveolar hemorrhage.

### Microglia Immunohistochemical Staining and Analysis

Immunohistochemistry was performed on 10-μm-thick sections from mouse brains. The sections were incubated overnight at 4 °C with 0.5 µg/mL of rabbit anti-mouse Iba1 (Wako). Biotinylated goat antirabbit (7.5 µg/mL; Vector Laboratories) was used as a secondary antibody. Iba1-immunopositive cells were visualized with DAB (3,3′-diaminobenzidine tetrahydrochloride) as previously described [34]. Sections stained with no primary antibodies were used as a control. Images were analyzed with Fiji software. Iba1-immunostained area was expressed as positive pixels per total assessed pixels and reported as the percentage of total stained area [35]. Microglia shape descriptor analysis was performed on images processed as previously described [36]. The cells were measured for area, perimeter, and Feret diameter (maximum caliper). Mean single-cell values for each parameter were used for statistics. Selectively in the brain cortex, 4 images at 20× with higher resolution were obtained to quantify the number of ramifications by means of the grid-crossing method [36]. For isolectin B4 (IB4) staining and analysis, 10-μm-thick sections from mouse brains were stained with *Griffonia simplicifolia* IB4 conjugated with Alexa 647, followed by 1 µg/mL of Hoechst nuclear staining as previously described [37].

### Immunohistochemistry of Lung Sections

Lung sections were heated to 65 °C and then incubated twice with xylene for 30 minutes at room temperature. The sections were then rehydrated by incubating with isopropanol at decreasing concentrations (100%, 96%, 80%, and 70% in water for 15 minutes at room temperature) and washed with PBS. Antigen retrieval was performed by incubating the sections with Tris-EDTA buffer (10mM Tris base, 1mM EDTA solution, 0.05% Tween 20, pH 9.0) at 95 °C for 30 minutes, followed by 3 washing steps with PBS. Nonspecific binding was reduced by blocking the lung sections with 10% donkey serum (30 minutes at room temperature). The sections were incubated for 1 hour with mouse IgG2b anti–SARS-CoV-2 NP (1:50 dilution; Fisher UK) and goat IgG anti-MASP2 (1:100 dilution; Santa Cruz). After another 3 washing steps with PBS (15 min/wash), the sections were incubated with Alexa Fluor 568–conjugated donkey anti-mouse IgG (1:200 dilution; Fisher UK) and/or Alexa Fluor 647–conjugated donkey anti-goat IgG (1:200 dilution; Fisher UK). After 3 washes in PBS, the sections were counterstained with DAPI (Abcam) diluted 1:5000 in PBS for 15 minutes at room temperature. Finally, the sections were washed with water and mounted with Vectashield Vibrance mounting medium.

## RESULTS

### LP Is Activated in the Lungs of SARS-CoV-2–Infected Mice

**Figure 1.**
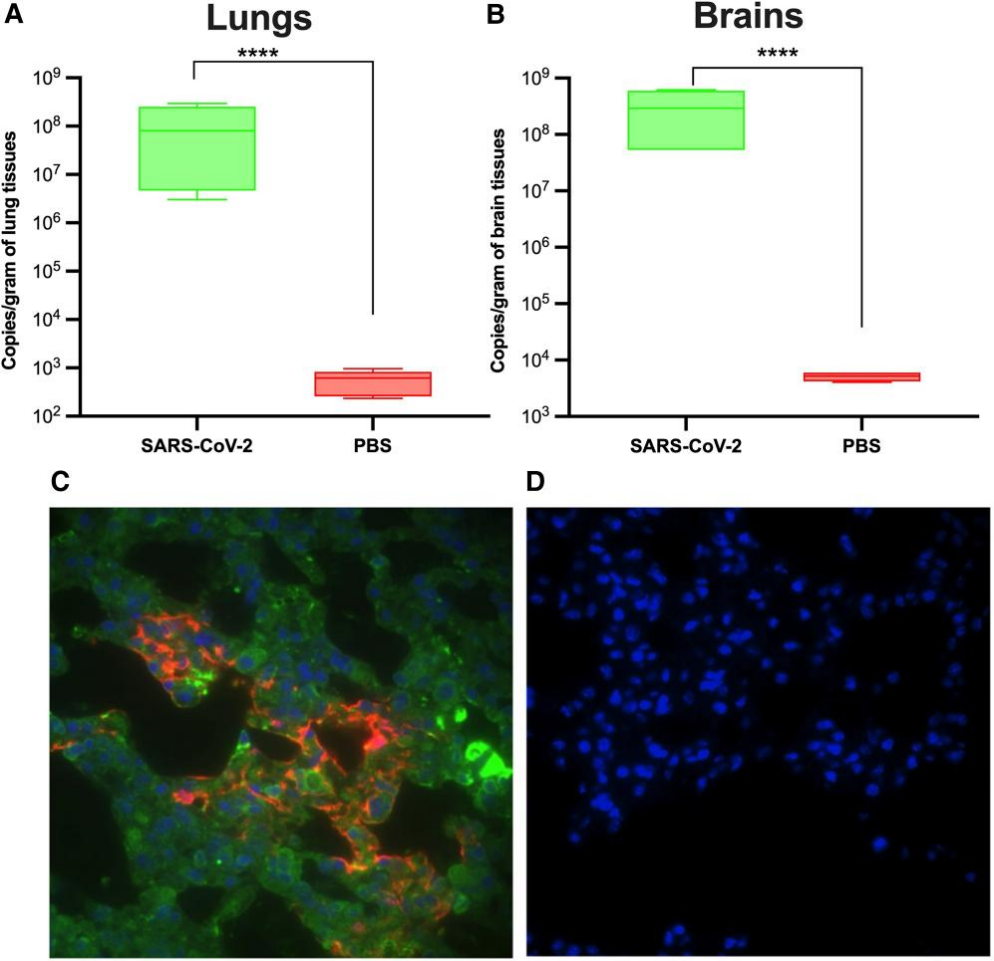
SARS-CoV-2 β variant infects k18-hACE-2 transgenic mice and expresses high levels of viral NP protein in lung tissues, which stimulates the lectin pathway. Twelve-week-old k18-hACE-2 transgenic mice were infected intranasally with SARS-CoV-2 β virus. Lungs and brains were collected 5 days postinfection. The copy numbers of viral transcripts in lung and brain tissue homogenates were measured by real-time reverse transcriptase–polymerase chain reaction. *A* and *B*, A significantly high level of viral RNA was detected in the lungs and brains of noninfected mice vs the lungs of noninfected mice (n = 5 mice). Data are mean ± SEM. (****P < .00001, Student *t* test). Lung sections of infected mice were stained with antibodies against SARS-CoV-2 NP proteins (red) and MASP-2 (green) and visualized by fluorescence widefield microscopy. High levels of NP and MASP-2 deposition were observed in lungs from infected mice (*C*) vs lungs from noninfected mice (*D*). Nuclei were stained with DAPI (blue).

To evaluate the ability of the SARS-CoV-2 β variant to infect humanized k18-hACE-2 mice, animals were challenged intranasally with 10^4^ plaque-forming units/40 μL of SARS-CoV-2 and monitored for signs of disease progression. Mice were euthanized 5 days postinfection, when the animals started to show symptoms of SARS-CoV-2 infection. A high viral load was detected in lung and brain tissues of infected mice (Figure 1*A*, *B*). In addition, high levels of viral NP and MASP-2 deposition were detected in lung tissues from infected mice as compared with noninfected lungs (Figure 1*C*, *D*), indicating activation of the LP of complement.

### Inhibition of the LP Reduces Mortality and Symptoms of Disease After SARS-CoV-2 Infection

**Figure 2.**
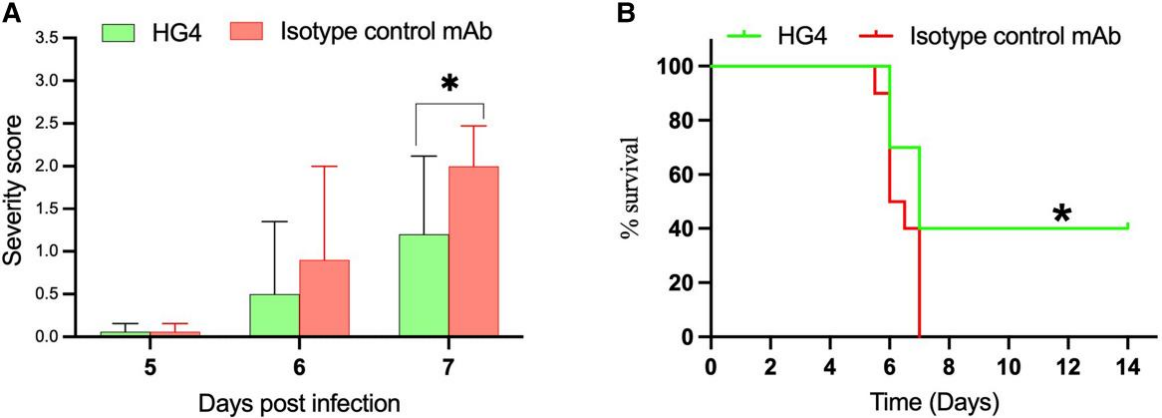
Administration of HG4 reduces the disease severity in mice after SARS-CoV-2 infection. Mice were infected intranasally with 10^4^ plaque-forming units/40 μL under light anaesthesia. Treated mice received HG4 (100 μg/animal) via intraperitoneal injection at days −1, 2, and 5 postinfection. Control mice received an isotype control monoclonal antibody. *A*, Mice treated with HG4 had significantly lower disease severity than mice that received control monoclonal antibody. Data are presented as mean ± SEM. n = 10/group. *P < .05, Student *t* test. Mice that received HG4 also had significantly increased survival time vs the control group (B). n = 10/group. *P < .05, Mantel-Cox log-rank test.

To assess the extent to which the LP inhibitor HG4 protects mice from the mortality associated with SARS-CoV-2 infection, 2 groups of k18-hACE-2 transgenic mice were intranasally infected with the SARS-CoV-2 β variant. Several days postinfection, the mice began to show signs of disease, such as hunched posture, labored breathing, and lethargy. Treatment of mice with HG4 improved the clinical scores and reduced the disease severity, as reflected by an improvement in breathing and activity and a reduction in neurologic symptoms, such as hunching and tremors, as compared with a control group treated with an isotype control antibody (*P* < .05; Figure 2*A*). At day 7 postinfection 100% of control mice reached the humane end point of disease progression and were euthanized, while 40% of HG4-treated mice survived the infection (*P* < .05; Figure 2*B*).

### Treatment With HG4 Protects Mice From Lung Injury due to SARS-CoV-2 Infection

**Figure 3.**
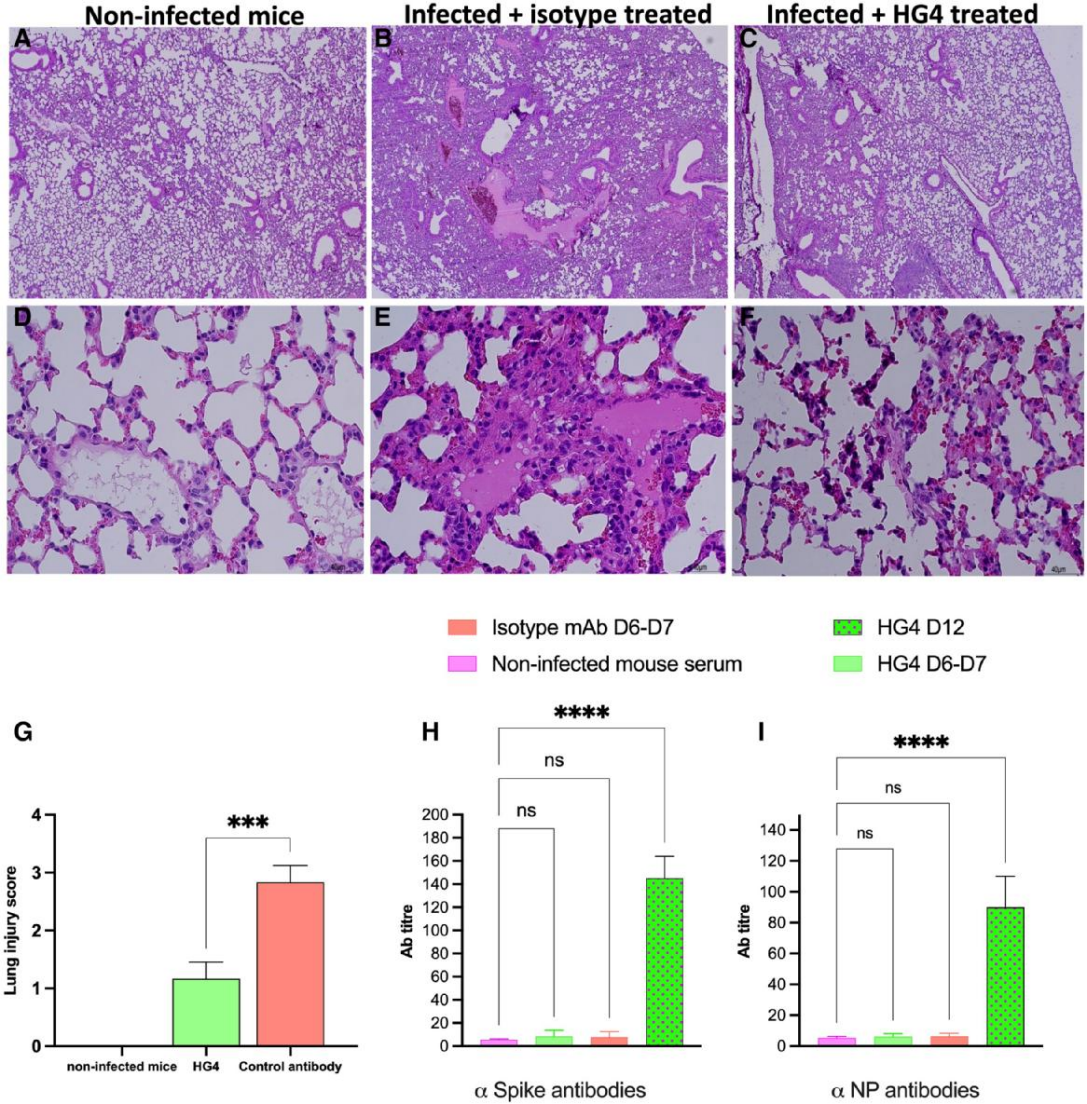
Inhibition of the lectin pathway in mice significantly reduces lung pathology after SARS-CoV-2 infection. Mice were treated with HG4 or an isotype control antibody 24 hours before infection and then on days 2 and 5 postinfection with SARS-CoV-2. Mice were observed and scored for clinical signs and disease progression. Lungs were collected at day 7 postinfection, and lung sections were prepared. Mice treated with HG4 (*C,F*) showed markedly less histopathologic evidence of lung damage (ie, interstitial alveolar inflammatory cell infiltration, thickening of interstitial membrane, acute intra-alveolar oedema, diffuse alveolar damage, and alveolar hemorrhage) than those treated with an isotype control antibody (*B,E*). Normal lung sections were used as a control (*A*,*D*). Magnification was 40× (*A*–*C*) and 400× (*D*–*F*). *G*, The lung injury score was significantly reduced in mice that received HG4 vs control. Results were analyzed with a Student *t* test. n = 4 lungs in each group. ***P < .001. *H*, *I*, Antibody titers against SP and NP were calculated in sera from infected mice. No specific antibodies were detected against SP or NP at 7 days postinfection while significantly high levels of antibodies were detected at 12 days postinfection (*H*,*I*). ****P < .0001. Data were analyzed by 1-way analysis of variance, followed by a Tukey test for multiple comparisons, and are presented as mean ± SEM.

Intranasal administration of SARS-CoV-2 to mice causes marked inflammation in the lung. This inflammation is mainly due to the influx of inflammatory cells into lung tissues, which induces lung injury. Mice treated with an isotype control antibody showed significant lung injury, including increased interstitial alveolar inflammatory cell infiltration, thickening of interstitial membranes, acute intra-alveolar edema, diffuse alveolar damage, and alveolar hemorrhage as compared with normal lungs from noninfected mice (Figure 3*A*, *B*, *D*, *E*). Mice that received HG4 had significantly reduced lung injury (Figure 3*C*, *F*) vs mice treated with an isotype control. The degree of lung injury was evaluated by a semiquantitative histopathologic score to assess the beneficial effect of HG4 in reducing lung pathology during SARS-CoV-2 infection. The average pathology score was significantly lower in mice treated with HG4 when compared with mice receiving an isotype control antibody (Figure 3*G*). To further investigate if anti–SARS-CoV-2 antibodies could drive complement activation via the CP and contribute to complement-mediated lung injury and general inflammatory pathology, we measured the levels of antibodies against SARS-CoV-2 in sera of infected mice. The antibody levels against SARS-CoV-2 SP and NP were below the lower limits of detection (giving only weak background signals as seen in sera of noninfected mice) on days 6 and 7 following SARS-CoV-2 infection, the time point at which all isotype-treated infected mice had to be culled due to the severity of murine COVID-19 symptoms. In contrast, strong antibody responses against SP and NP were detected in sera of surviving HG4-treated SARS-CoV-2–infected mice at 12 days postinfection (Figure 3*H*, *I*).

### Inhibition of the LP Significantly Reduces Brain Inflammation During COVID-19 Pathogenesis in Mice

**Figure 4.**
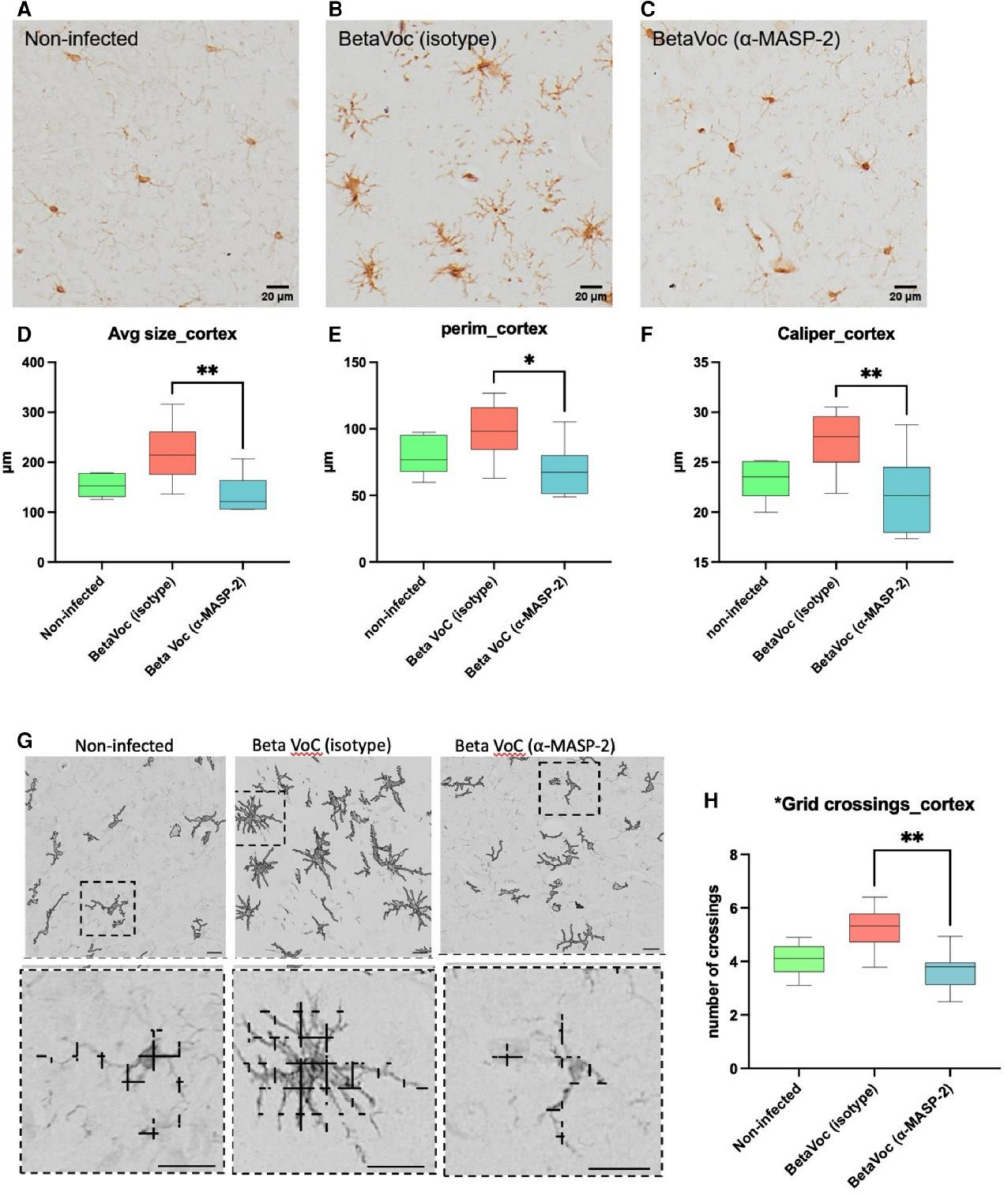
Inhibition of MASP-2 significantly reduces brain inflammation in response to SARS-CoV-2 infection. Mice were given either the MASP-2 inhibitor HG4 or an irrelevant isotype control antibody 24 hours before infection and on days 2 and 5 postinfection with SARS-CoV-2. Brains were harvested 7 days postinfection. Brain sections were prepared and stained immunohistochemically with an Iba1 antigen–specific antibody to identify brain myeloid cells. The microglial phenotype in the cortical brain sections of mice treated with the MASP-2 inhibitor HG4 (*C*) is very similar to that of homeostatic microglia in noninfected brains (*A*), while microglial cells in the cortex of SARS-CoV-2–infected mice treated with an irrelevant isotype control antibody (*B*) show the typical hypertrophic shape of reactive microglia. Microglia shape description analysis revealed that microglial cells from mice treated with HG4 have significantly less average size (*D*), perimeter (*E*) and Feret diameter (*F*) vs isotype control antibody–treated animals. *G*, Micrographs of microglial ramifications showed that Iba1^+^ cells from mice treated with HG4 have less ramified morphology than cells from nontreated animals. The upper panel shows segmented Iba1^+^ cells, while the lower panel shows magnification of the crossings of the segmented signal. *H*, Grid-crossing analysis demonstrates that Iba1^+^ cells from infected mice receiving the control antibody display a significantly increased presence of ramified microglia with more frequent crossings with the grid as compared with microglia from mice that received HG4. Data were analyzed by 1-way analysis of variance, followed by a Tukey test for multiple comparisons. *P < .05, **P < .01. Scale bars 20 µm. VoC, variant of concern.

**Figure 5.**
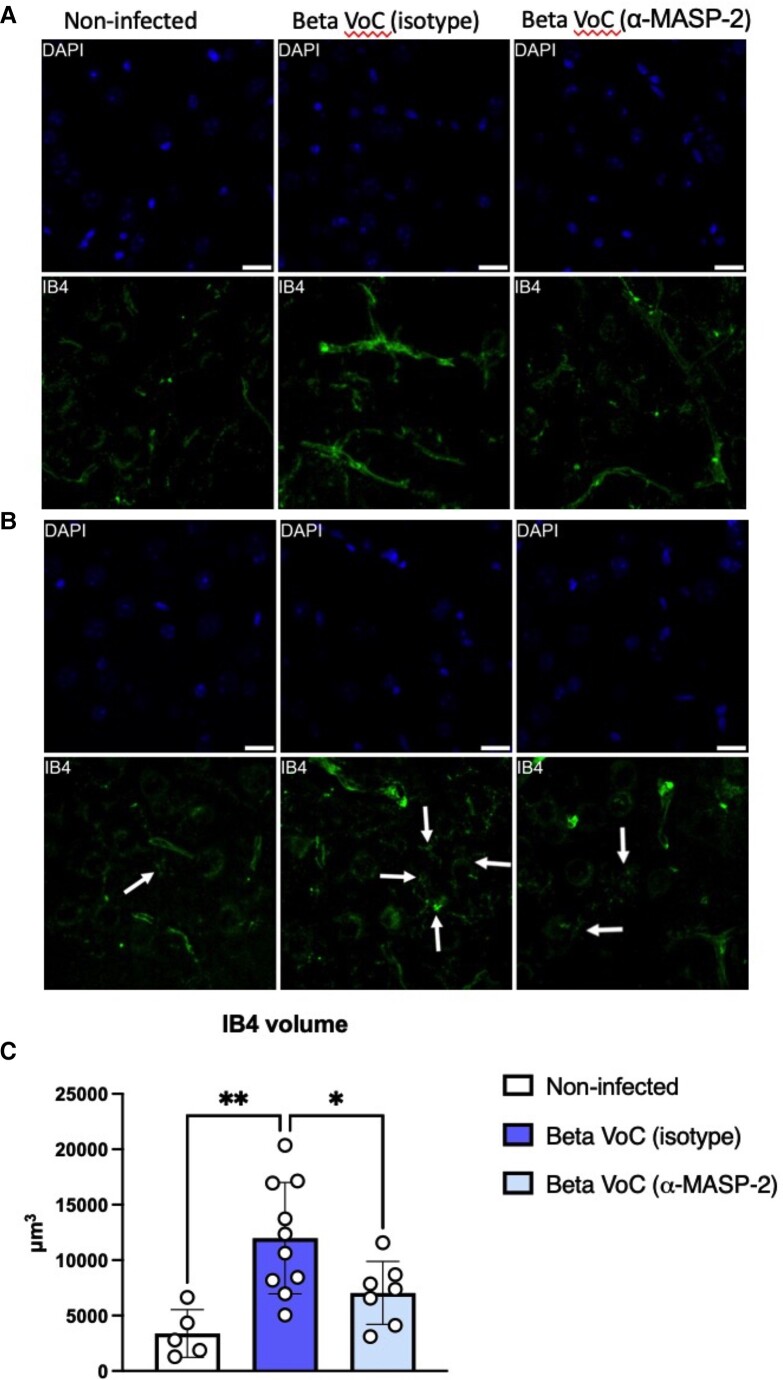
Inhibition of MASP-2 significantly reduces isolectin B4 staining in response to SARS-CoV-2 infection. Brain sections of SARS-CoV-2–infected mice treated with either HG4 or an isotype control antibody were stained with fluorescent IB4 (green) and compared with stained sections from noninfected mice. Nuclei were stained with DAPI (blue). *A*, The relative volume of IB4-stained (green) endothelial and perivascular tissue. *B*, The various stages of IB4-positive microglial cells stained with different intensity (green; see arrows). The intensity of IB4 staining was significantly reduced in mice treated with HG4 vs mice that received an isotype control antibody only. *C*, The intensity of IB4 staining positively correlates with the inflammatory activation of IB4-positive cells; for comparison, see the relative quantification of the IB4 signals (mean ± SEM). Brain sections from noninfected mice were used as controls. Scale bars = 20 µm. Data were analyzed by 1-way analysis of variance, followed by a Tukey test for multiple comparisons. *P < .05. **P < .01. VoC, variant of concern.

Given the known neurologic symptoms associated with COVID-19, we chose to determine whether the anti-inflammatory effects of MASP-2 inhibition extend beyond the lung to the CNS. Microglial activation was measured in the brains of SARS-CoV-2–infected mice treated with either HG4 or an irrelevant isotype control antibody. Brain sections from infected animals were stained with Iba1 as a marker for brain myeloid cells. Treatment of infected mice with HG4 reduced, in the brain cortex, the stained positive area of Iba1 vs mice treated with an isotype antibody (mean ± SD; area fraction, 2.34 ± 1.82 vs 6.00 ± 3.34; Figure 4*A*–*C*). Since microglia morphology is indicative of microglial activation, data from cell-shape descriptor analysis showed that hypertrophic/hyperactivated morphology of microglia is clearly visible after SARS-CoV-2 infection. When infected mice were treated with HG4, there was a significant reduction in the average area (135.2 ± 38.6 vs 221.5 ± 56.5 µm^2^), perimeter (68.8 ± 20.2 vs 99.2 ± 19.7 µm), and Feret diameter (caliper, 21.8 ± 4.1 vs 27.1 ± 2.8 µm) of the microglia as compared with mice treated with the control antibody (Figure 4*D*–*F*). With a more detailed analysis based on fully resolved microphotographs, the density of microglia ramifications was quantified by the grid-crossing method—specifically, counting the number of contacts between each cell and an overimposed grid with 9-µm spacing as previously described [38]. Treatment with HG4 reduced microglia complexity by lowering the number of grid contacts (ie, ramifications) when compared with mice treated with control antibody (Figure 4*G* and 4*H*). To assess if HG4 treatment can also ameliorate blood vessel inflammation and decrease the inflammatory response in the infected brains, we stained sections of the mouse brains with IB4, which stains carbohydrate residues in the glycocalyx of cells in a proinflammatory state, including endothelial cells, perivascular neurons, and perivascular microglia [39, 40]. In line with this, the IB4 signal was associated with brain vessels and ramified perivascular cells. The volume occupied by the IB4-positive signal was markedly reduced when infected mice were treated with HG4 vs mice treated with an isotype control antibody (Figure 5).

## DISCUSSION

SARS-CoV-2 infection elicits a wide spectrum of symptoms in humans, ranging from the total absence of any signs of disease to mild-moderate disease and life-threatening inflammatory conditions caused by overshooting inflammatory responses to the virus, which can lead to ARDS and death [41]. A strong association has been demonstrated between the degree of complement activation and the severity of endothelial damage in the lung during SARS-CoV-2 ARDS. Analysis of lung sections from patients who died from SARS-CoV-2 infection showed an extensive deposition of complement C4d and MASP-2, suggesting ongoing activation of the LP [42].

In this study, we used a model of SARS-CoV-2 infection in k18-hACE-2 mice to assess the therapeutic utility of complement inhibition targeting the LP effector enzyme MASP-2. Following intranasal infection with the β variant of SARS-CoV-2, all isotype control–treated mice developed the hallmarks of severe acute COVID-19 and had to be culled 7 days after infection. Inhibition of the LP with HG4 significantly decreased the clinical signs and symptoms of disease progression and mortality in SARS-CoV-2–infected mice. Lung histology presented in this work showed the beneficial effect of LP inhibition in reducing the lung pathology associated with ARDS throughout SARS-CoV-2 infection. Treatment with anti-MASP-2 antibody significantly reduces leukocyte infiltration and inflammatory exudates in the lungs of HG4-treated animals as compared with isotype control–treated animals, resulting in marked improvement in lung pathology and lung injury score. In the brain, MASP-2 inhibition reduces local inflammation, as suggested by a decrease in microglial reactivity and volume of tissue stained by IB4. These results broadly agree with the outcome of a previous clinical study where the anti–MASP-2 antibody narsoplimab was evaluated as a potential treatment for patients with acute severe COVID-19 ARDS. Narsoplimab was shown to improve lung function and laboratory markers and reduce overall disease severity and mortality [28]. All patients in this study likely had SARS-CoV-2–specific antibodies, as the detection of covalently bound C1s/C1Inh complexes indicates that the CP was activated [26]. It is unknown at present to what extent CP-driven activation of complement by SARS-CoV-2–specific antibodies contributes to COVID-19 pathology [26]. As inhibition of MASP-2 functional activity alone achieved significant recovery from hypocomplementemia, the LP and not the CP appears to be primarily responsible for the massive consumption of complement components in patients with acute COVID-19. Narsoplimab treatment in these patients resulted in rapid inhibition of LP functional activity and reduced levels of the AP activation product Bb, while CP-mediated complement activation was unaffected. We therefore conclude that LP activation is synergistically amplified through the AP amplification loop in acute severe COVID-19 [28].

The absence of detectable antibody responses to SARS-CoV-2 infection in k18-hACE-2 mice within the first 7 days sheds further light on the critical contribution of LP-driven complement activation in the early stages of COVID-19. As signs of murine COVID-19 developed within the first week after SARS-CoV-2 infection leading to terminal inflammatory pathology, the absence of antibodies against SARS-CoV-2 within this time frame implies that activation of complement through anti–SARS-CoV-2 antibodies via the CP is unlikely to account for the early onset of severe inflammatory pathology in COVID-19. In contrast, at day 12 following infection, the surviving HG4-treated mice had high titers of antibodies against SARS-CoV-2 proteins that could drive complement activation via the CP. However, there is no evidence for a second wave of CP-driven inflammatory lung injury at that stage. Previous reports have shown that in human serum, the LP is highly activated at an early stage and throughout the course of COVID-19 infection [39, 43]. A previously published study excluded CP activation at an early stage of infection where C1q binding to the SARS-CoV-2 SP or NP was not detected, while both viral proteins bound to the LP recognition subcomponents MBL, CL-11, and FCN2 [43]. The findings of the present mouse study strongly support the predominant role of the LP over the CP in driving complement-dependent inflammatory pathology in response to SARS-CoV-2 infection because the massive activation of complement in the early stages of COVID-19 occurs during the absence of SARS-CoV-2–specific antibodies. We therefore postulate that the LP is the key initiator of complement activation in response to SARS-CoV-2 infection and is supported by the amplification of complement activation through the AP.

SARS-CoV-2 infects multiple other tissues in the body besides the lung, with the endothelium and the CNS being of particular interest because of the known cardiovascular and neurologic symptoms. In this study, we focused on microglia, the brain’s resident macrophages, which act as a nonneuronal innate sensor against viral infection [38]. Once activated, microglia transform from less to more ramified morphology, releasing several inflammatory cytokines, such as interleukins 1β and 6 and tumor necrosis factor α, which participate in the induction of a robust inflammatory response and cytokine storm in the CNS [18].

Microglia reactivity is a typical hallmark of brain injuries and is associated with neuroinflammation and lesion expansion. Microglia constitutively express complement receptors; thus, their activation is driven by complement products, including those downstream to MASP-2. Our results demonstrate that treatment of SARS-CoV-2–infected mice with HG4 significantly reduced the level of microglial activation and the volume of IB4-stained tissues when compared with mice treated with control antibodies. The use of HG4 was reported to attenuate microglia reactivity in a mouse model of acute ischemic stroke [16]. The volume of IB4-stained tissues is another marker that correlates with the degree of brain inflammation and breakdown of the brain-blood barrier [40].

Given its mechanism of action, it is unlikely that HG4 treatment had any effect on the virus itself but rather on the MASP-2–driven proinflammatory activity in response to the virus. A shift in microglial activation toward hypertrophic reactive microglia might lead to neurotoxicity and other CNS deleterious effects [44, 45]. The prevention of that shift to activation, as seen with MASP-2 inhibition, could be beneficial in ARDS as well as a range of CNS disorders associated with microglial activation. The recent observation that the inhibition of MASP-2 functional activity significantly protects from LPS-induced ARDS [46] implies that MASP-2 is critically involved in the development of inflammatory lung pathology independent of the initial driver of lung inflammation.

## Notes

***Acknowledgments.*** We thank Adrian Stover and Aurora Bianchi (Mario Negri IRCCS) and Galina Gragerova and Heidi Utsugi (Omeros Corporation) for providing excellent technical support.

***Financial support.*** This work was supported by the National Institute for Health and Care Research/UK Research and Innovation (NIHR/UKRI) (grant number COV0170, Humoral Immune Correlates of COVID-19, to W. S.); the Omeros Corporation, Seattle, USA; Wellcome Trust Senior Research Fellowships (106207/Z/14/Z, 220814/Z/20/Z to H. S.); the European Research Council (to H. S. by grant 646891 awarded to Prof Andrew Firth, University of Cambridge); and Innovative UK DIOS-CoVax (72845 DiosynVax to G. W. C., C. L. G., and J. L. H.).
